# New Gene Markers Involved in Molecular Processes of Tissue Repair, Response to Wounding and Regeneration Are Differently Expressed in Fibroblasts from Porcine Oral Mucosa during Long-Term Primary Culture

**DOI:** 10.3390/ani10111938

**Published:** 2020-10-22

**Authors:** Artur Bryja, Patrycja Sujka-Kordowska, Aneta Konwerska, Sylwia Ciesiółka, Maria Wieczorkiewicz, Dorota Bukowska, Paweł Antosik, Rut Bryl, Mariusz T. Skowroński, Jędrzej M. Jaśkowski, Paul Mozdziak, Ana Angelova Volponi, Jamil A. Shibli, Bartosz Kempisty, Marta Dyszkiewicz-Konwińska

**Affiliations:** 1 Department of Anatomy, Poznan University of Medical Science, 60-781 Poznań, Poland; artekbr@o2.pl (A.B.); rutbryl@gmail.com (R.B.); m.dyszkiewicz@ump.edu.pl (M.D.-K.); 2 Department of Histology and Embryology, Poznan University of Medical Science, 60-781 Poznań, Poland; psujka@ump.edu.pl (P.S.-K.); akonwer@ump.edu.pl (A.K.); sciesiolka@ump.edu.pl (S.C.); 3 Department of Anatomy and Histology, University of Zielona Gora, 65-046 Zielona Góra, Poland; 4 Department of Basic and Preclinical Sciences, Institute of Veterinary Medicine, Faculty of Biological and Veterinary Sciences, Nicolaus Copernicus University in Torun, 87-100 Toruń, Poland; maria.wieczorkiewicz@umk.pl (M.W.); skowron@uwm.edu.pl (M.T.S.); 5 Department of Diagnostics and Clinical Sciences, Nicolaus Copernicus University in Torun, 87-100 Toruń, Poland; dbukowska@umk.pl (D.B.); jmjaskowski@umk.pl (J.M.J.); 6 Department of Veterinary Surgery, Nicolaus Copernicus University in Torun, 87-100 Toruń, Poland; pantosik@umk.pl; 7 Physiology Graduate Program, North Carolina State University, Raleigh, NC 27695, USA; pemozdzi@ncsu.edu; 8 Department of Craniofacial Development and Stem Cell Biology, King’s College University of London, London WC2R 2LS, UK; ana.angelova@kcl.ac.uk; 9 Department of Periodontology and Oral Implantology, Dental Research Division, University of Guarulhos, Guarulhos SP 07030-010, Brazil; jashibli@yahoo.com; 10 Department of Obstetrics and Gynecology, University Hospital and Masaryk University, 601 77 Brno, Czech Republic; 11 Department of Biomaterials and Experimental Dentistry, Poznan University of Medical Sciences, 61-701 Poznań, Poland

**Keywords:** primary culture, porcine, fibroblasts, microarray

## Abstract

**Simple Summary:**

Wound healing and vascularization mechanisms are key steps in the complex morphological process of tissue reconstruction. Additionally, these processes in the oral cavity are more rapid than in the skin and result in less scar formation. Epithelial cells and fibroblasts play an important role in the process of wound healing. In our study, we focused on fibroblasts and monitored changes in gene expression during their in vitro culture. Based on the analysis, we distinguished three groups of processes that play important roles in tissue regeneration: response to wounding, wound healing and vascularization. We identified genes that were involved in all three processes. These genes could be selected as tissue specific repair markers for oral fibroblasts.

**Abstract:**

The mechanisms of wound healing and vascularization are crucial steps of the complex morphological process of tissue reconstruction. In addition to epithelial cells, fibroblasts play an important role in this process. They are characterized by dynamic proliferation and they form the stroma for epithelial cells. In this study, we have used primary cultures of oral fibroblasts, obtained from porcine buccal mucosa. Cells were maintained long-term in in vitro conditions, in order to investigate the expression profile of the molecular markers involved in wound healing and vascularization. Based on the Affymetrix assays, we have observed three ontological groups of markers as *wound healing group*, *response to wounding group* and *vascularization group*, represented by different genes characterized by their expression profile during long-term primary in vitro culture (IVC) of porcine oral fibroblasts. Following the analysis of gene expression in three previously identified groups of genes, we have identified that transforming growth factor beta 1 (*TGFB1*), *ITGB3*, *PDPN*, and *ETS1* are involved in all three processes, suggesting that these genes could be recognized as markers of repair specific for oral fibroblasts within the porcine mucosal tissue.

## 1. Introduction

The proper course of wound healing needs several specific processes that occur in a particular order. The first phase is initiated by mesenchymal cells migrating to the wounded area. In the next steps, angiogenesis and epithelial processes are required. Then, the following steps take place—regeneration and fibrosis processes. Regeneration includes events related to the replacement of damaged cells by the same type of cells [1]. Fibrosis is a process in which damaged tissue is replaced by connective tissue [1]. These processes occur in epithelial cells as well as in fibroblasts, which can replicate at a high rate under stimulation [2].

When we search for an attractive source of cells that could be used in regenerative therapeutic approaches, this source could be cells isolated from oral mucosa which is an easily accessible tissue characterized by the capacity of fast scarless wound-healing [3].

The oral mucosa undergoes continuous modifications [4,5,6], requiring maintenance of homeostasis between cell survival and apoptosis, which substantially influence the cellular composition of oral tissue and change ionic balance [7,8]. Oral tissue must be able to quickly adapt to environmental events and due to its properties, it is the subject of multiple avenues of research. Molecular events during the healing process in oral mucosa have been extensively studied and the differences have been observed in scarless wound healing (present in fetal skin tissues) and in the production of inflammatory triggered extracellular matrixes within adult tissue [9,10,11,12,13,14].

Few comprehensive transcriptome studies of oral mucosa have been reported. Chen et al. compared the expression profiles between skin and oral mucosal tissue in a wound-healing experimental mouse model, highlighting the difference in the levels of proinflammatory cytokines that were far less produced in the oral mucosa, compared to skin [15]. In another study, Enoch et al. showed that the wound stimuli induced cell proliferation and re-organization of the collagenous environment much more pronouncedly in human oral fibroblasts, than in human skin fibroblasts [2]. This noticeable increased proliferation ability of buccal mucosal cells was demonstrated in our recent studies [16]. Parallel to cell proliferation, the wound healing process encompasses the induction of tissue vascularization and neoangiogenesis [17,18].

In our study, we focus on fibroblasts isolated from porcine mucosa. It is proved that fibroblasts play an important role in wound healing [2,19,20,21]. The porcine oral mucosa has similar properties to human mucosa. There are studies that confirm more rapid wound healing in oral mucosa than in skin and that the formation of granulation tissue composed of fibroblasts in an injury place is faster in oral mucosa than in skin [22]. Moreover, it has been shown that porcine oral mucosal wounds demonstrate more similar molecular composition and histological similarity with the scar compared to human oral mucosal wounds [20].

We performed a gene profile expression analysis of three important ontogenic groups, (hallmarks of tissue repair), at three-time points in long term culture conditions of porcine oral fibroblasts. The relevance of the study captures the important aspect of investigating the gene profile signature of these cells in primary culture as well as sheds light on the dilemma of maintaining these cells properties in vitro for a long time.

## 2. Materials and Methods

### 2.1. Animals

Sixty pubertal crossbred Landrace gilts were bred on a local commercial farm, with a mean age of 155 days (range 140–170 days), and the mean weight was 100 kg (95–120 kg). Tissue collection was performed following the slaughter of the animals in a commercial slaughterhouse. No animals were sacrificed for research purposes only. The current research was accepted by Local Animal Ethics Committee of the Poznan University of Life Sciences, Poland (permission no. 32/2012, 30.06.2012).

### 2.2. Histological Analysis

Immediately after slaughter, 10 samples of the buccal mucosa and buccal muscle were immersed in Bouin’s solution for 48 h. Afterwards, the collected tissues were dehydrated and embedded in paraffin blocks. In the next stage, the paraffin blocks were cut with a semi-automatic rotary microtome (Leica RM 2145, Leica Microsystems, Nussloch, Germany), and all paraffin sections were stained with standard hematoxylin and eosin (H&E) staining method. In the first step, the paraffin sections were deparaffinized and rehydrated. After this step, the samples were stained with hematoxylin and eosin. In the end, the sections were dehydrated and coverslipped. Subsequently, histological sections were assessed with a light microscope, and selected pictures were taken using a scanning technique by Olympus BX61VS microscope scanner (Olympus, Tokyo, Japan).

### 2.3. Tissue Preparation and Cell Culture

**Figure 1:**
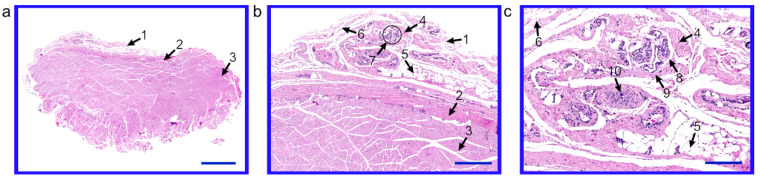
The porcine buccal tissue samples stained with hematoxylin and eosin (H&E) method. (**a**) lamina propria of the mucosa, fascia and buccal muscle, scale bar length: 2000 µm, (**b**) lamina propria of the mucosa, fascia and buccal muscle, scale bar length: 500 µm, (**c**) lamina propria of the mucosa, scale bar length: 200 µm. 1—lamina propria of the mucosa, 2—dense connective tissue of fascia, 3—skeletal muscle, 4—dense connective tissue of mucosa, 5—adipose tissue, 6—loose connective tissue, 7—blood vessels, 8—arterioles, 9—venules, 10—circulatory anastomoses.

Tissue samples of buccal mucosa were transported to the laboratory in previously prepared sterile transportation media (0.9% NaCl supplemented with 10 mg/mL streptomycin, 10 U/mL penicillin G, and 25 µg/mL amphotericin B). In the laboratory, the tissue samples were washed in Dulbecco’s phosphate buffered saline (D-PBS; Sigma Aldrich, Madison, WI, USA). The buccal epithelium was surgically separated from the underlying lamina propria and dense fascia connective tissue using a sterile surgical blade. For the in vitro culture, lamina propria, dense and loose connective tissue of the mucosa was used (Figure 1). After preparation, separated tissue was minced using surgical blades. In order to isolate fibroblasts, digestion in a collagenase solution was used [23,24]. Therefore, fragments of tissue (approximately 0.5 mm^3^) were incubated with 0.05% collagenase I (Sigma Aldrich, Madison, WI, USA) for 40 min at 37 °C in a shaking water bath. After digestion, the cell suspension was filtered through a mesh to obtain a single-cell suspension. The solution with cells was centrifuged for 10 min at 200× *g* to obtain a cell pellet. Cells were resuspended in Dulbecco’s modified Eagle’s medium (DMEM; Sigma Aldrich, Madison, WI, USA) supplemented with 10% FCS, 10 U/mL penicillin G, 10 mg/mL streptomycin, and 25 µg/mL amphotericin B. The viability and number of cells obtained was determined using a cell counter Adam-MC (NanoEnTek, Seoul, Korea).

**Figure 2:**
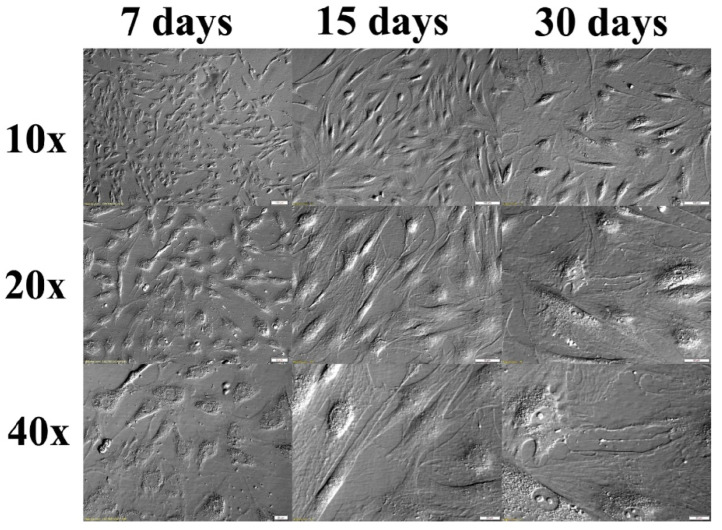
Cells morphology during long-term in vitro culture (IVC). Nomarski phase/contrast images. Scale bars length for magnification 10×: 100 µm, 20×: 50 µm, 40×: 20 µm. Cells present elongated bipolar or stellate shapes that are characteristic for fibroblasts.

The cells for one in vitro culture were pooled from tissue acquired from 10 pigs. The isolated cells were seeded at a density of 2 × 10^4^ cells/cm^2^ and the viability was not less than 90%. The oral fibroblasts were cultured at 37 °C in a humidified atmosphere of 5% CO_2_. The culture medium was changed every three days. Cells were passaged when they attained 70–80% confluency. Before the collection of cells for the analyzed samples, photos of the culture were taken to observe the possible changes of morphology. Observations were documented at three-time points: 7 days, 15 days, and 30 days (Figure 2). For further experiments, 5 in vitro cultures were performed. Cells from two cultures were used for microarray analysis and cells from three cultures were used for Quantitative Reverse Transcription PCR (RT-qPCR).

### 2.4. Microarray Expression Analysis and Statistics

During long-term in vitro culture, material was collected for RNA isolation in 7, 15 and 30 day intervals. For each interval two samples delivered from two in vitro cultures were collected. Total RNA was extracted from cells using the Chomczynski and Sacchi method [25]. RNA quantity and purity were determined spectrophotometrically at 260 and 280 nm wavelengths (Epoch, Biotek, Bad Friedrichshall, Germany). For each sample two rounds of sense cDNA amplification (Ambion^®^ WT Expression Kit, Waltham, MA, USA) were conducted. In the next steps cDNA was used for biotin labeling and fragmentation by Affymetrix GeneChip^®^ WT Terminal Labeling and Hybridization (Affymetrix, Thermo Fisher Scientific, Waltham, MA, USA). Biotin-labeled fragments of cDNA (5.5 μg) were hybridized to the Affymetrix^®^ Porcine Gene 1.1 ST Array Strip (48 °C/20 h). Washing and staining were conducted by employing the Affymetrix GeneAtlas Fluidics Station. The array strips were scanned using the Imaging Station of the GeneAtlas System (Waltham, MA, USA). Analysis of the scanned chips was made using Affymetrix GeneAtlasTM Operating Software (Waltham, MA, USA).

The obtained results were analyzed using Bioconductor version 3.11 (open source project, http://www.bioconductor.org/) and R version 3.6.3 programming language (free software, http://www.r-project.org/) The Robust Multiarray Averaging (RMA) algorithm was used for normalization and summarization of the results. To determine the statistical significance of the analyzed genes, moderated t-statistics from the empirical Bayes method were performed. The obtained *p* value was corrected for multiple comparisons using Benjamini and Hochberg’s false discovery rate. The selection of significantly changed genes was based on a *p* value lower than 0.05 and expression higher than two-fold.

The differentially expressed gene list (separated for up- and down-regulated genes) was uploaded to DAVID software (Database for Annotation, Visualization and Integrated Discovery) [26], where genes belonging to *wound healing*, *response to wounding* and *vasculature development* gene ontology (GO) terms were obtained. The relation between those genes were checked with the GOplot R package [27].

Interactions between differentially expressed genes/proteins belonging to the *wound healing*, *response to wounding* and *vasculature development* gene GO terms were investigated by STRING version 10 software (Search Tool for the Retrieval of Interacting Genes) (open source project, http://string-db.org/) [28]. The list of gene names was used as a request for an interaction prediction. The search criteria were based on co-occurrences of genes/proteins in scientific texts (text mining), co-expression, and experimentally observed interactions. The results of such analyses generated a gene/protein interaction network where the intensity of the edges indicated the strength of the interaction score.

### 2.5. Validation of Microarray Analysis Using Real-Time Quantitative Polymerase Chain Reaction (RT-qPCR)

Material for analysis was collected from in vitro cultured cells. RNA was isolated at intervals of 7, 15 and 30 days. For each interval three samples were collected from three in vitro cultures. Total RNA was extracted using the Chomczynski and Sacchi method [25]. RNA quantity and purity were tested with a spectrophotometr (Epoch, Biotek, Bad Friedrichshall, Germany). RT-qPCR was carried out using LightCycler (Roche Diagnostics GmbH, Mannheim, Germany). Detection of cDNA was evaluated by employing SYBR^®^ Green I. Target cDNA was calculated using the relative quantification method. ACTB and HPRT were used as the internal standards, and analyzed genes were calculated in each sample to these housekeeping genes. The amplification process was carried out by adding 2 µL of cDNA solution to 18 µL of QuantiTect^®^ SYBR^®^ Green PCR (Master Mix Qiagen GmbH, Hilden, Germany) and primers (Table 1). One RNA sample of each mixing was processed as a negative control. The results were calculated using the 2^-^^ΔΔCT^ method described by Livak and Schmittgen [29].

## 3. Results

In the histological analysis, the collected porcine buccal tissue samples stained with the H&E method have revealed the proper morphology with the presence of lamina propria of the mucosa, fascia and buccal muscle. The epithelium was difficult to show due to its significant mobility within the cheek. The mucosa was mainly composed of connective tissue, both dense and loose. Adipose tissue was also visible. Small blood vessels of the nature of arteries and veins, as well as circulatory anastomoses were also observed. The dense connective tissue was separating the mucosa and buccal muscle from the fascia. The buccal muscle was composed of skeletal muscle (Figure 1).

To investigate porcine oral fibroblasts transcriptome changes between 7 (zero passage), 15 (one passage) and 30 days (two passages) from the start of primary cell culture, we performed whole gene expression analysis by Affymetrix^®^ Porcine Gene 1.1 ST Array (data are available in Gene Expression Omnibus (GEO) database, accession number: GSE112659). Expression of more than 15,809 porcine transcripts was investigated. The genes for which the fold change was higher than the absolute value of two (fold > |2|) and the *p* value was lower than 0.05, were deemed as differentially expressed. From these genes 38 were upregulated and 44 were downregulated between day 7 and 15 of culture, 52 were upregulated and 26 were downregulated between the 7th and 30th day of culture, and 13 were upregulated and one was downregulated between day 15 and 30 of culture (Figure S1).

**Figure 3:**
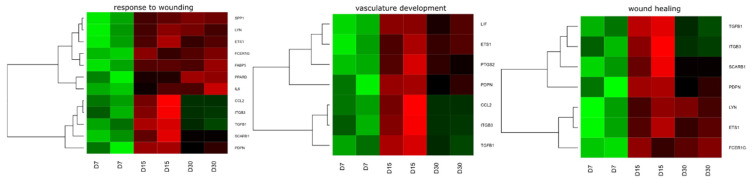
Heat map representation of differentially expressed genes belonging to the *wound healing*, *response to wounding* and *vasculature development* gene ontology (GO) terms. Arbitrary signal intensity acquired from microarray analysis is represented by colors (green, higher; red, lower expression).

**Figure 4:**
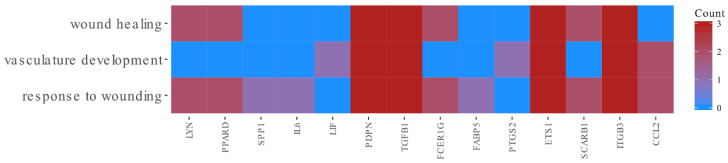
Heatmap showing the gene occurrence *wound healing*, *vasculature development* and *response to wounding* GO terms. The presented count value means the number of gene occurrence in the analyzed GO terms.

**Figure 5:**
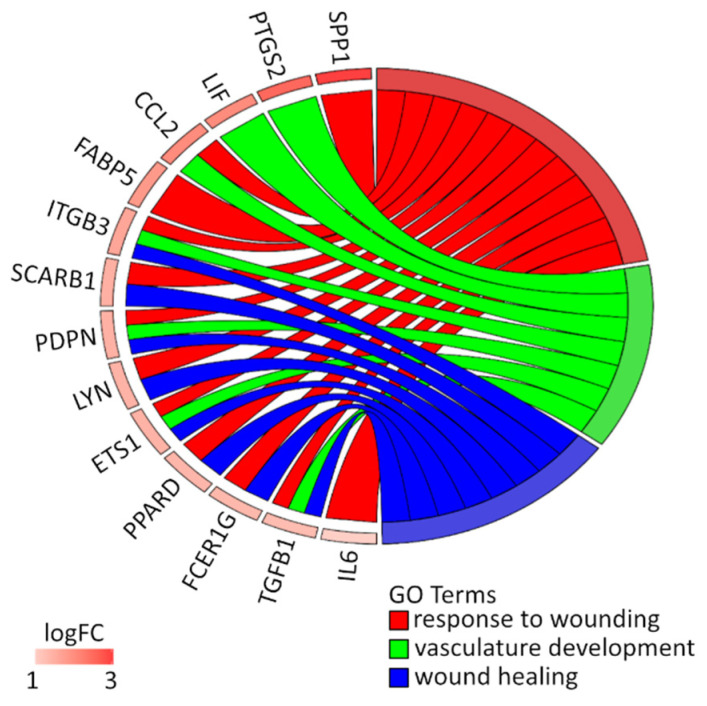
The correlation between differentially expressed genes that belongs to the *wound healing* (blue ribbon), *response to wounding* (red ribbon) and *vascular development* (green ribbon) GO terms. The ribbons join categories with the genes shared with each other. The genes were sorted by log_2_(fold change) from most to least changed gene.

Among these genes, genes from *wound healing*, *response to wounding* and *vasculature development* GO groups were extracted by DAVID (Database for Annotation, Visualization and Integrated Discovery) software. These sets of genes were subjected to a hierarchical clusterization procedure and presented as heatmaps (Figure 3 and Figure 4). The differently expressed genes that are divided between those GO terms are presented in Figure 5 and Table 2. In the Supplementary Materials, all of the differentially expressed genes are presented in a table (Table S1) and table with a list of all ontological groups obtained during the microarray analysis (Table S2)

To further discover known interactions between genes of interest, a STRING-generated interaction network was generated between differentially expressed genes belonging to *wound healing* (the sequence of events that restore integrity to a damaged tissue, following an injury, GO:0042060), *response to wounding* (any process that results in a modification inactivity of a cell as a result of a stimulus indicating damage of the organism, GO:0009611) and *vasculature development* (the process whose specific outcome in the development of the vasculature over time, from its formation to the mature structure, GO:0001944) gene ontology (GO) terms. Employed prediction methods that used text mining, co-expression, experimentally observed interactions (Figure S2).

**Figure 6:**
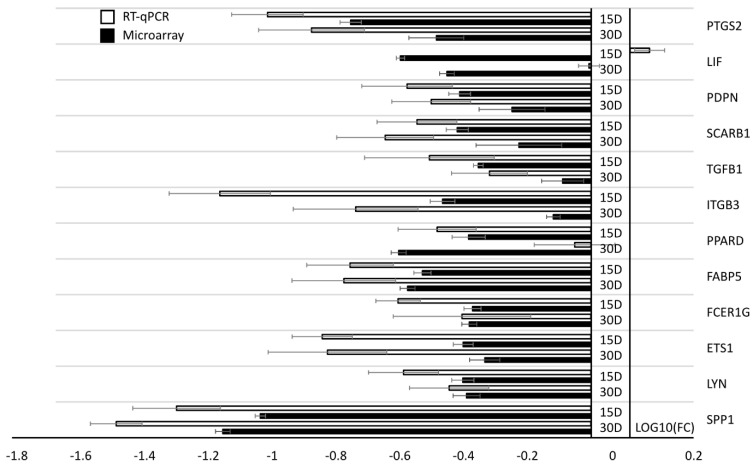
Graphic representation of RT-qPCR validation for selected genes that were included in the microarray analysis. The presented data were compared to the 7th-day of in vitro culture. Presented data are ±SD of values obtained from n = 2 (microarray: black bars) and n = 3 (RT-qPCR: white bars) of biological replicates.

RT-qPCR analysis was performed in order to quantitatively validate the results of the microarray analysis, with the results shown in the form of Figure 6. The analyzed genes were down-regulated compared to expression on day 7. The direction of changes in gene expression were validated for all analyzed genes except *LIF*. *LIF* did not confirm microarray changes in direction at comparison of day 30 to day 7.

## 4. Discussion

Oral mucosa, with its unique scarless and fast wound healing capabilities [30], is a suitable model for research into repair and regeneration mechanisms. The oral mucosa in pigs has similar properties to human and constitutes a convenient model for various avenues of research. Therefore, as a study model, we have chosen porcine oral mucosa where similarities in thickness and keratinization, as well as expression of antigens for p63 and PCNA (markers for epithelial stem/progenitor cells), DNA replication and proliferation are relevant to humans [5]. The expression of stem/progenitor cell markers in mucosal cells during their culture suggests that these cells maintain large differentiation capability and stem-like plasticity in vitro [31,32].

Wound healing is an inherent response to injury, resulting in the restoration of tissue integrity. It is often described as a series of events, resulting in gene transcription and protein translation that leads to the main stages of wound healing. Epithelial cells and fibroblasts play an inseparable role in this process. The progenitor/stem-cell-enriched population was successfully isolated from cultured primary oral mucosal keratinocytes [33]. This is one of the cell populations that affects the ability of oral tissue to heal without scar in a fetal-like manner [34]. Fibroblasts constitute another cell population within the oral mucosa that are shown to play a role in the wound healing process. Interactions between epithelial cells and connective tissue cells are extremely important for the proper development of epithelial tissue [35]. Fibroblasts also seem to be important in that they may have a similar expression pattern to mesenchymal cells [36]. As Van Wyk et al. showed, the doubling times of oral fibroblasts was shorter than dermal fibroblasts, however, there was no observed statistical significance [37]. Similar data were presented by Mah et al. They described that fibroblasts isolated from the gingiva were characterized by a faster proliferation rate compared to fibroblasts isolated from the skin. Additionally, gingival fibroblasts presented a higher level of expression of genes involved in the regulation of inflammation and remodeling of the extracellular matrix [38].

In this study we have analyzed the expression levels of selected groups of genes, involved in different aspects of wound healing in porcine oral fibroblast primary culture. We have investigated oral fibroblasts transcriptome changes between days 7 (zero passage), 15 (one passage) and 30 (two passages) in culture. We have focused on three gene ontology groups: *response to wounding*, *vasculature development* and *wound healing*. Capturing the regenerative potential of cells isolated from oral mucosa in an in vitro setting would be highly beneficial for future translational studies. We have observed that genes from all three analyzed groups presented highest expression levels on day 7 and then a significant decrease in expression was observed on day 15 and 30.

One of our hypotheses was that the mechanism of upregulation resembles the in vivo reaction to injury. The creation of new tissue relates to the second stage of wound repair and occurs between 2 and 10 days after injury [39]. New granulation tissue will begin to form beneath the epithelium approximately four days following injury. Fibroblasts are the major components that supply the new extracellular matrix. Together with blood supply they sustain the forming tissue’s metabolism. A general hypothesis regarding the series of events leading to angiogenesis, is that injury leads to hypoxia and releases angiogenic factors. It is well known that transforming growth factor beta (TGFB) plays a very important role in scarring. TGFB is a trigger for the reorganization of the actin cytoskeleton [40]. Reduced or more transient expression of TGFB and its receptors has been found in non-scarring fetal wounds as compared to adult wounds. In a study by Szpaderska et al. [41,42] significantly lower levels of interleukin 6 and interleukin 8 were shown, during 72-h period, in mucosa versus skin. A similar situation was observed for TGFB1. We have observed increased expression of TGFB1 on day 7, which agreed with the observation of Murphy-Ullrich et al. [40]. TGFB1 plays an important role in the maintenance of tissue homeostasis by regulating cell growth, differentiation and extracellular matrix accumulation. TGFB1 transgene stimulation in oral mucosa results in increased angiogenesis, and subsequent epithelial hyperproliferation [43]. TGFB1 is produced in a latent form, bound to a latent TGF-B1-binding protein-1 (LTBP-1), and can be activated by plasmin and modulated by fibromodulin. The regulation of TGFB1 activation may also play a role in modulating repair. Since the TGFB superfamily proteins and related signaling transduction pathways are significantly involved in the regulation of cellular growth, proliferation and differentiation, we suggested that greater expression of TGFB1 at 7 day of in vitro culture (IVC) may be associated with an increased cell proliferation index and with reaching the confluency of cells in vitro. An increase in TGFB expression may also have an effect on increased collagen production by fibroblasts during in vitro culture [19].

The increased expression of genes associated with the wound response, angiogenesis and healing suggests reinforced regeneration potential of our primary cell cultures on day 7, with a slow decrease in its expression, reaching a significant drop on days from 15 to 30. Although the results suggest a similar pattern of gene expression for in vitro cultures and in vivo reactions to injury, the gene downregulation, observed at later time points of culture, could be associated with the duration and conditions of cell culture (in vitro). This may be due to in vitro culture conditions. The applied culture medium promoted the growth of fibroblasts. The other cell types present in the studied culture encountered significant culture stress, which could have inhibited their proliferation and at further time intervals, when the in vitro culture became stabilized, only fibroblasts remained. Januszyk et al. [44], in their study, investigated the transcriptional effects of passaging a human stem cell population prior to gene expression profiling. Their results showed that cells undergo substantial transcriptional changes during cell culture from the time of cells seeding and after the first passage. They also observed a significant change in the dynamics of the subpopulation of cells, with gene expression profiles having similar properties to stem-like cells. This conclusion may contribute to the explanation of our findings.

In 2015 Miyoshi K. et al. [45] analyzed the gene signature of human oral mucosa fibroblasts, in comparison with dermal fibroblasts and induced pluripotent cells. Their findings revealed that tissue-reconstructive, proliferative, and signaling pathways are active, whereas senescence-related genes in p53 pathway are inactive in human oral fibroblasts (hOFs). Furthermore, more than half of hOF-specific genes were similarly expressed to those of hOF-iPSC genes and might be controlled by WNT signaling.

Our results suggest higher expression of several genes that are involved in all three ontological groups (Figure 5). As shown in previous studies, the main difference between healing processes in the oral cavity and the skin is quickly resolving inflammation in oral mucosa wounds compared with long-lasting inflammation in the wounded skin [46]. In support of this statement, Szpaderska et al. [42] confirmed the presence of fewer inflammatory cells in oral wounds, supported by the measurement of decreased cytokine production. Decreased production of *IL-6* and the platelet-derived growth factor-inducible KC (*KC*) in oral mucosal wounds may be responsible for the reduced recruitment of neutrophils and macrophages. They also observed differences in the expression of *TGFB1*, a pro-inflammatory, pro-fibrotic cytokine that has been found in hypertrophic scars [47]. This is in accordance with our results where we observed an increase in the expression of *TGFB1*, that plays an important role in wound healing and repair as a key regulator of the production and remodeling of the extracellular matrix (ECM), through its effect on mesenchymal cells, shown to be maintained in our long-term cultured porcine oral mucosa cells.

An even more interesting result, was that parallel to the expression of *TGFB1*, we observed a high expression of ETS proto-oncogene 1 (*ETS1*), a factor that regulates matrix turnover by activating several metalloproteinases (MMPs) [48]. In previous studies it has been shown that *ETS1* strongly suppresses *TGFB* induction of collagen type 1 and other matrix-related genes and reverses TGFB-dependent inhibition of MMP-1 [48]. Our results suggest that the high level of expression of ETS1 in cultured porcine oral fibroblasts is maintained, suggesting that ETS1 could still be actively involved as an effector of the TGFB signaling pathway and act as an antagonist of the profibriotic effects of TGFB. It is of great importance profiling the characteristics of in vitro-maintained (cultured) cells of the oral mucosa, that can potentially be used in future translational studies of repair and regeneration.

An analysis comparing gene expression in dermal fibroblasts and gingival fibroblasts was performed by Ebisawa et al. [49]. Using microarrays, they analyzed the expression of 5284 genes, and they showed that only 5% of these genes had a significant difference in the expression level. Three genes were confirmed with the most significant difference: *IGF2*, *HSD17B2*, and *GPC3*. However, we have not identified common genes in our research. Ebisawa et al. compared the gene expression of skin fibroblasts to gingival fibroblasts, and in our study, we have compared the expression of buccal mucosa fibroblasts at different time intervals.

In conclusion, our results suggest that porcine oral fibroblasts present the expression of important genes responsible for basic wound healing mechanisms, as illustrated by the three heatmaps grouping genes involved in response to wounding, vasculature development, and wound healing processes. In the wound healing process, the appropriate modulation of the extracellular matrix, which does not lead to scarring, is of great importance. The *TGFB* and *ETS* genes seem to be important for this process. The properties of in vitro cultured cells may resemble the properties of oral mucosa as a tissue and therefore in vitro cultured cells may be the basis for research into the scar-free wound healing process. However, despite the current discoveries, more research in this area is required before translating our knowledge into clinical therapeutic approaches.

## Figures and Tables

**Table 1:** Oligonucleotide sequences of primers used for RT-qPCR analysis.

Gene	Entrez Gene ID	Primer Sequence (5′-3′)	Product Size (bp)
ETS1	100302363	atcagctggacaggagatgg gtttacccgccgtcttgtg	166
FABP5	574074	aagaatgggacgggaaggag ttcatagacccgagtgcagg	104
FCER1G	397406	accctcctctactgtcgact ataagtctcctggttccggg	111
ITGB3	397063	ctcatcggccttgctactct agagacacccacaatcctgg	231
LIF	399503	gtgccaacgccctctttatt attgaggctcctttggtccc	209
LYN	100152890	agaggccatcaacttcggat tctgcaggtagtcgaaggtg	248
PDPN	100738269	ggtgcaatcatcgtcatgct ttccacgggtcatcttctcc	158
PPARD	397590	caatgccctggaactcgatg ttgatccgctgcatcatctg	249
PTGS2	397018	aaaggcctcaatcgaccaga atctgggcgaggcttttcta	202
SCARB1	397087	ccccatcgtctaccagatcc agtcctgaagaagtggggtg	242
SPP1	397078	agaagttccgcagatccgaa tccgtctcctcactttccac	82
TGFB1	100302363	accatgccaatttctgcctg gaacgcacgatcatgttgga	208
ACTB	414396	cccttgccgctccgccttc gcagcaatatcggtcatccat	69
HPRT	397351	ccatcacatcgtagccctc acttttatatcgcccgttgac	171

**Table 2:** Fold-changes, adjusted *p* values and the Entrez gene ID of differentially expressed genes belonging to the *wound healing*, *response to wounding* and *vasculature development* gene ontology (GO) terms. Symbols and names of the selected genes are also shown.

Gene	LOG10(FC) D15/D7	LOG10(FC) D30/D7	*p* Value D15/D7	*p* Value D30/D7	Entrez Gene ID
CCL2	−0.59	−0.16	0.0407	0.4044	397422
ETS1	−0.40	−0.33	0.0311	0.0475	100302363
FABP5	−0.53	−0.57	0.0268	0.0235	574074
FCER1G	−0.37	−0.38	0.0268	0.0236	397406
IL6	−0.25	−0.35	0.0885	0.0442	107652590
ITGB3	−0.46	−0.12	0.0389	0.4214	397063
LIF	−0.60	−0.45	0.0128	0.0230	399503
LYN	−0.40	−0.39	0.0346	0.0423	100152890
PDPN	−0.41	−0.25	0.0342	0.1033	100738269
PPARD	−0.38	−0.60	0.0520	0.0235	397297
PTGS2	−0.75	−0.48	0.0333	0.0862	397590
SCARB1	−0.42	−0.23	0.0346	0.1343	397018
SPP1	−1.04	−1.15	0.0163	0.0230	397087
TGFB1	−0.35	−0.09	0.0150	0.2166	397078

## Supplementary Materials

The following are available online at https://www.mdpi.com/2076-2615/10/11/1938/s1, Figure S1: The volcano graph showed the process of selecting genes for which the levels of expression were significantly different in the analyzed time intervals. Differentially expressed genes were confirmed when the *p* value was less than 0.05 and the expression was higher than two-fold. Figure S2: STRING-generated interaction network among differentially expressed genes belonging to the *wound healing*, *response to wounding* and *vasculature development* GO terms. Applied prediction methods: text mining, co-expression, experimentally observed interactions. Table S1: List of differentially expressed genes obtained during the microarray analysis. Table S2: List of ontological groups obtained during the microarray analysis.
