# Supplementary figures and images for: New Gene Markers Involved in Molecular Processes of Tissue Repair, Response to Wounding and Regeneration Are Differently Expressed in Fibroblasts from Porcine Oral Mucosa during Long-Term Primary Culture
Source: Animals (Basel). 2020 Oct 22;10(11):1938. doi: 10.3390/ani10111938 (PMC7690285; doi:10.3390/ani10111938)

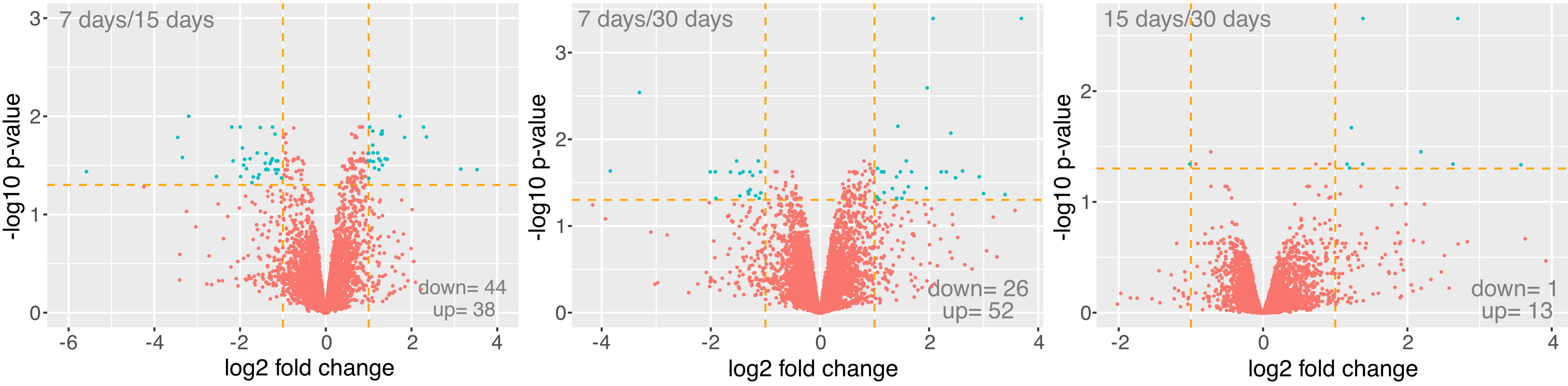

Supplement: Supplementary file 1 [file animals-10-01938-s001.zip › Bryja.Fig.S1.tiff]

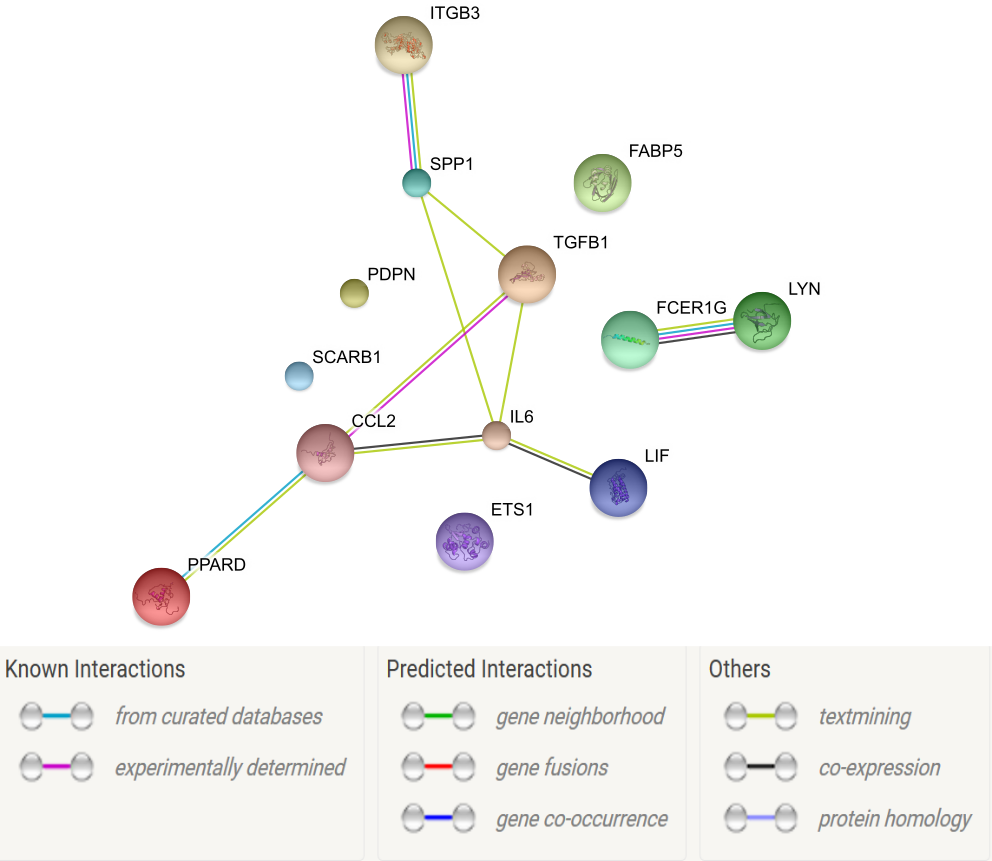

Supplement: Supplementary file 1 [file animals-10-01938-s001.zip › Bryja.Fig.S2.tiff]
